# Long‐Term Effects of Lecanemab on Biomarkers of Neurodegeneration in Plasma

**DOI:** 10.1002/alz.091966

**Published:** 2025-01-09

**Authors:** Charlotte Teunissen, Brian A. Willis, Pratik Bhagunde, Robert Bell, Natasha Penner, Pallavi Sachdev, Larisa Reyderman

**Affiliations:** ^1^ Neurochemistry Laboratory, Department of Clinical Chemistry, Vrije Universiteit Amsterdam, Amsterdam UMC location VUmc, Amsterdam, North Holland Netherlands; ^2^ Neurochemistry Laboratory, Department of Laboratory Medicine, Amsterdam Neuroscience, Amsterdam UMC, Vrije Universiteit Amsterdam, Amsterdam Netherlands; ^3^ Eisai Inc., Nutley, NJ USA

## Abstract

**Background:**

Lecanemab, a novel humanized immunoglobulin G1 monoclonal antibody targeting both neurotoxic Aβ protofibrils and Aβ plaques, has demonstrated the ability to substantially reduce markers of amyloid and significantly slow clinical decline on multiple measures of cognition and function in early AD in phase 2 (Study 201) and phase 3 (Clarity AD) studies. In these clinical studies, several plasma biomarkers assessments (Aβ42/40 ratio, p‐tau181, GFAP, and p‐tau217) showed improvements comparing lecanemab with placebo. Herein, we utilized modelling and simulations to evaluate the long‐term effects of lecanemab on biomarkers of neurodegeneration in plasma.

**Method:**

Models describing the relationship between serum lecanemab exposure and change in plasma biomarkers were developed using data pooled from lecanemab phase 2 and 3 studies. Individual serum lecanemab exposure was estimated using a population pharmacokinetic model and correlated with plasma biomarker concentrations using indirect response models. Simulations were conducted to evaluate the effect of lecanemab (10 mg/kg IV every 2 weeks) after 4 years of continuous treatment, discontinuation after 18 months of treatment, or transitioning to less frequent dosing at 18, 24, or 30 months.

**Result:**

Data were available for up to 48 months continuous lecanemab treatment (Clarity AD) or 18 months continuous treatment, followed by a pause in dosing (average 24 months, range 5‐59 months) followed by a resumption of dosing (Study 201). All models were well parameterized and adequately described the data. Simulations demonstrated that biomarkers reverted towards pre‐treatment baseline after cessation of lecanemab treatment, with an average half‐life of approximately 1‐1.5 years. Transitioning to less frequent monthly dosing was sufficient to stabilize plasma biomarker concentrations at levels consistent with lecanemab efficacy.

**Conclusion:**

Models describing the change in plasma biomarker levels over time in response to lecanemab treatment have been developed. Simulations conducted with these models demonstrate that continued treatment with lecanemab is required to sustain the benefit of lecanemab on amyloid and neurodegenerative pathology, but that less intensive dosing regimens are sufficient to maintain the changes in plasma biomarker levels consistent with lecanemab efficacy.

